# Supplementary material for: The Transcriptome of Chicken Migratory Primordial Germ Cells Reveals Intrinsic Sex Differences and Expression of Hallmark Germ Cell Genes
Source: Cells. 2023 Apr 13;12(8):1151. doi: 10.3390/cells12081151 (PMC10136783; doi:10.3390/cells12081151)
Supplement: Supplementary file 1 [file cells-12-01151-s001.zip › Supp Figures_Cells.pdf]

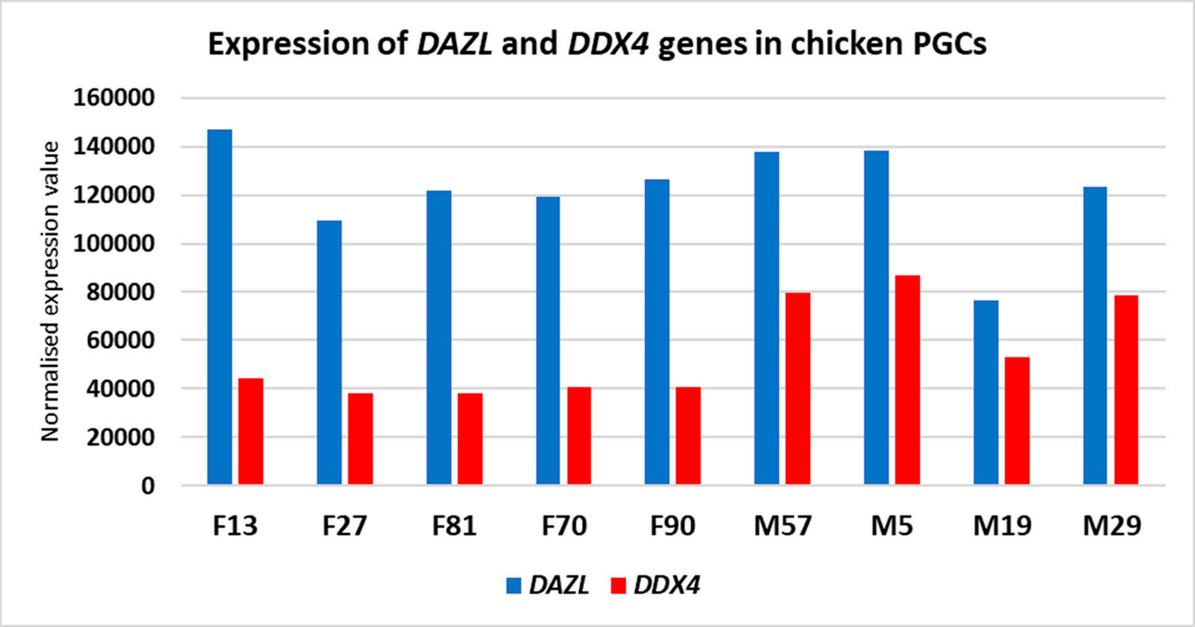

Supplementary Figure S1: Expression of germ cell-specific genes *DAZL* and *DDX4* in chicken PGCs. The normalized expression value to a gene was obtained from the DESeq2 package.

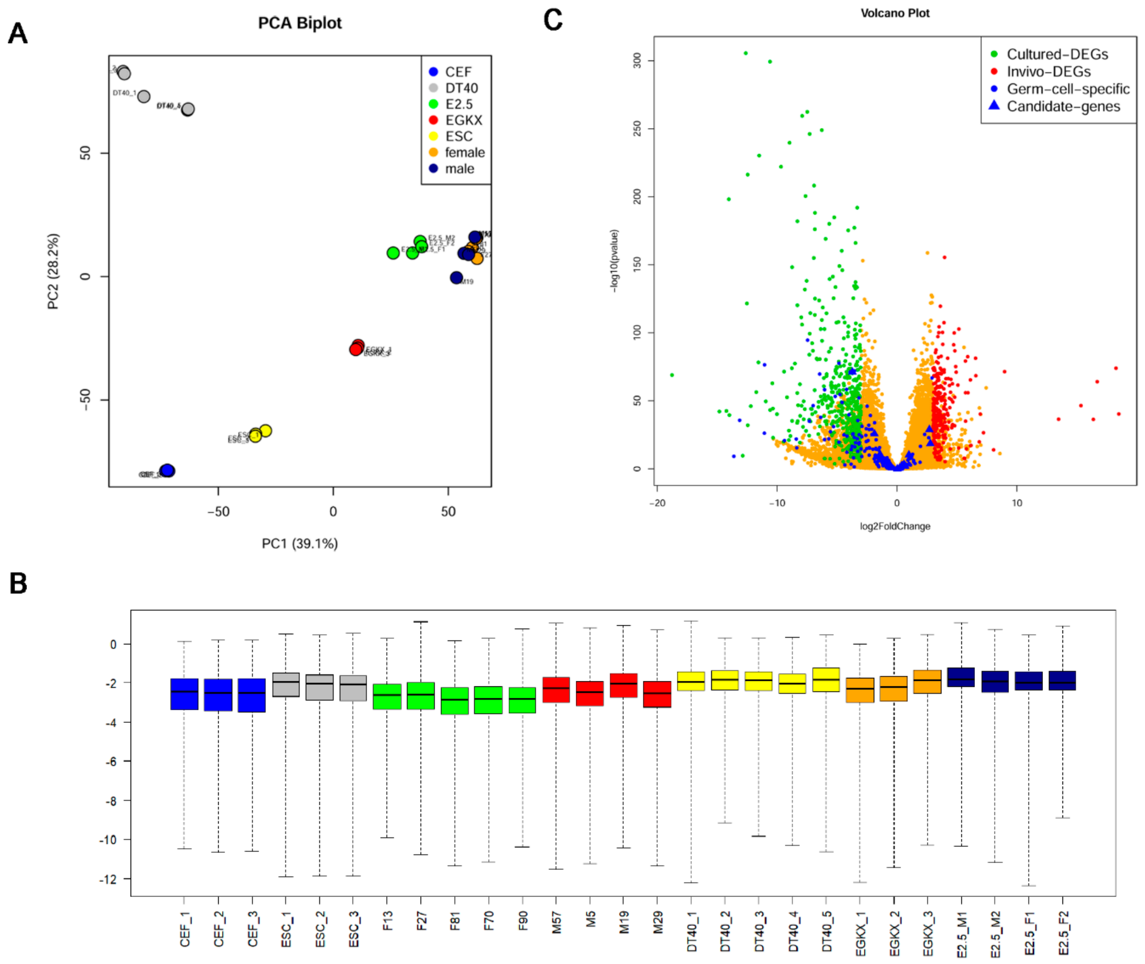

Supplementary Figure S2: A. PCA plot shows clustering of samples based on the similarity of the transcriptome profile (top 1000 variable genes) of chicken PGC lines using transcriptomic data of PGCs, *in ovo* PGCs(E2.5), chicken embryonic fibroblasts (CEF), DT40 cell line (DT40), embryonic stem cells (ESCs) and blastodermal cells (EGKX cells) from the public domain. **B.** Boxplot of Cooks distances shows that all samples are consistent and there are no outliers samples. **C.** Volcano plot showing differential expressed genes between cultured and *in ovo* PGCs (E2.5.)

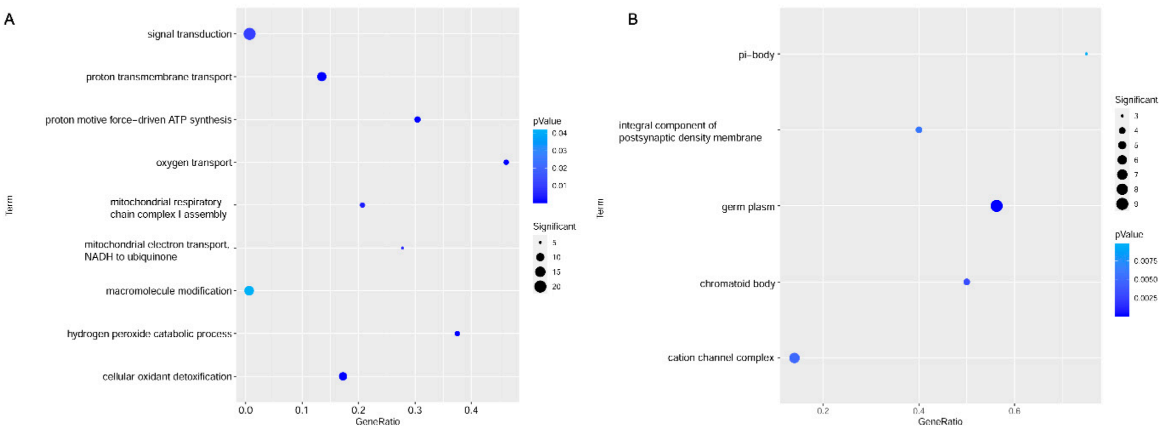

Supplementary Figure S3: A. Gene enrichment analysis (biological process) of genes expressed higher in *in ovo* PGCs. **B.** Gene enrichment analysis of germ cell exclusive genes in cellular components.

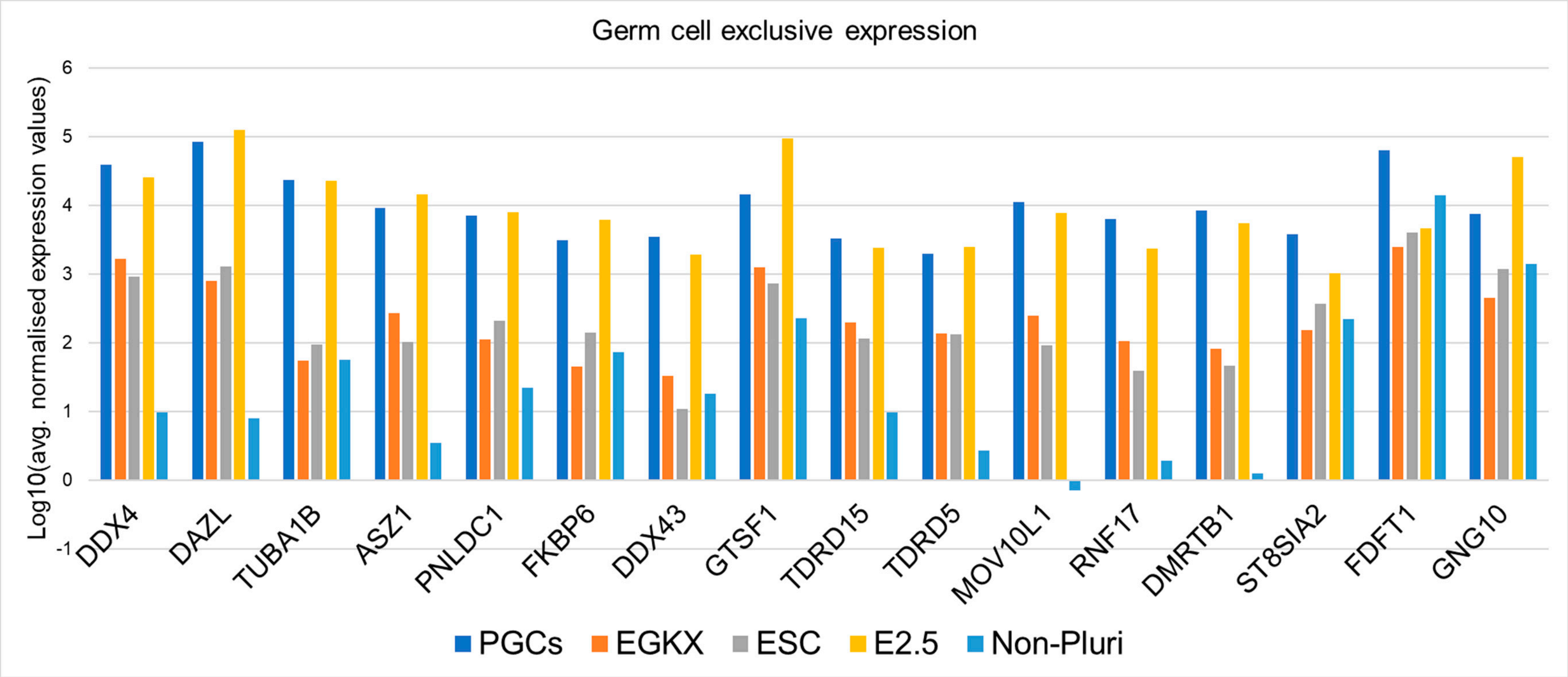

Supplementary Figure S4: Barplot showing the average expression of candidate genes that are exclusive to the germ cell. The normalized gene expression values calculated from the DESeq2 package for chicken PGCs, chicken laid egg stage, *in ovo* PGCs (E2.5) and other cell types such as ESC, CEF, and DT40, have been used to identify these candidate genes. The average expression from non-germ cell types is < 700 and the ratio of avg. expression values between PGCs and EGKX higher than ten were used to shortlist these genes. The cell lines CEF and DT40 are together referred as Non pluripotent (NonPluri) cells.

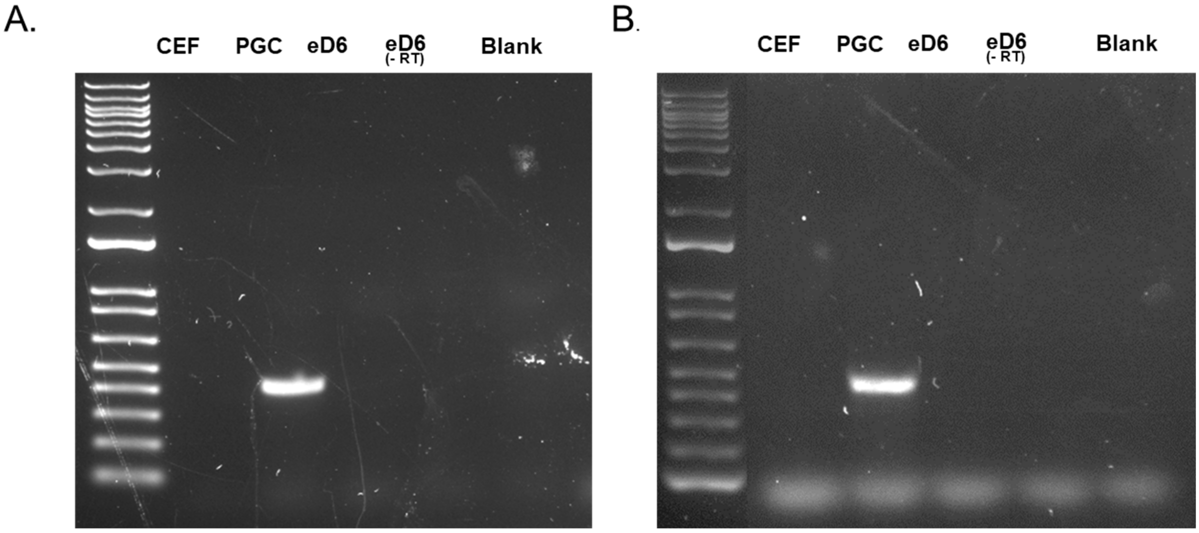

Supplementary Figure S5: The RT-PCR analysis of *DAZL* (A) and *DDX4* (B) genes in chicken samples. The expected product size for *DAZL* is 455bp and was found only in the PGC sample. Similarly, only PGCs show the expression of the *DDX4* gene whose product size is approximately 406bp. These were sequenced to verify the PCR product.

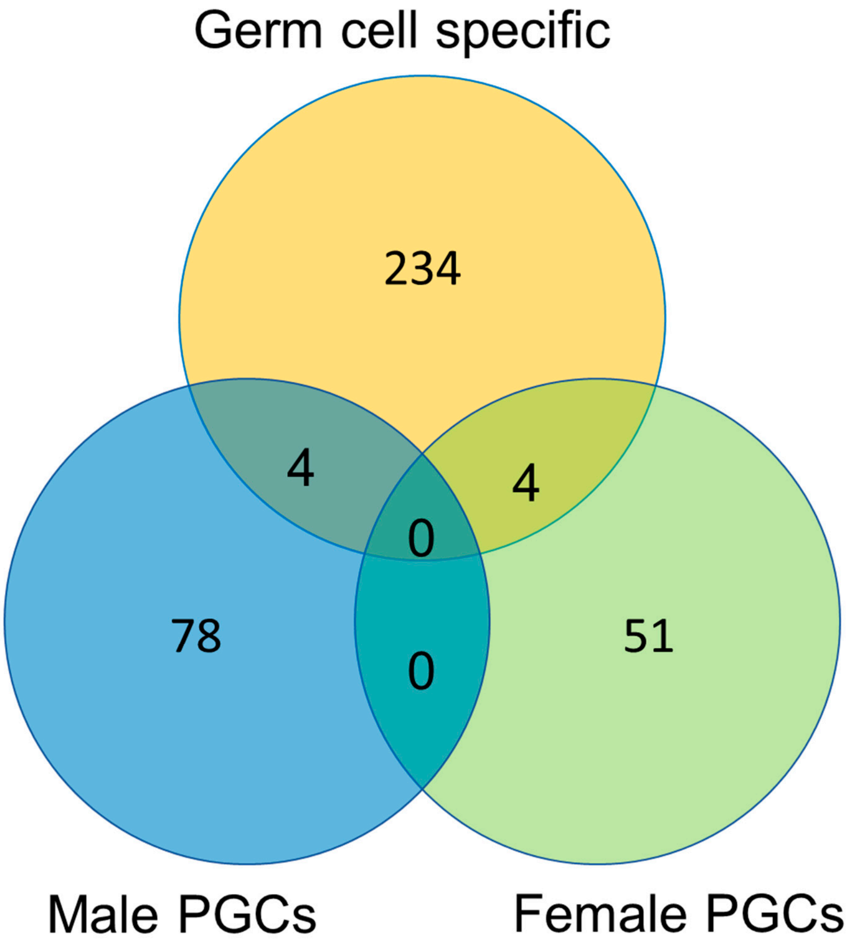

Supplementary Figure S6: Venn diagram showing overlapping male and female DEGs with germ cell exclusive genes.

A

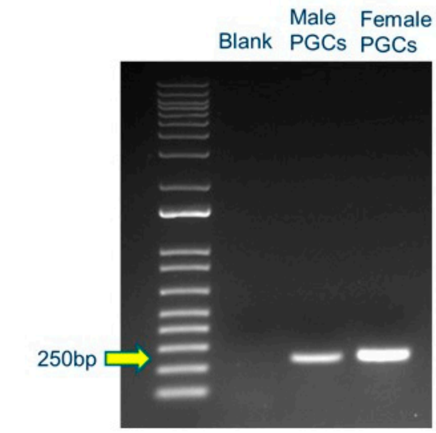

B

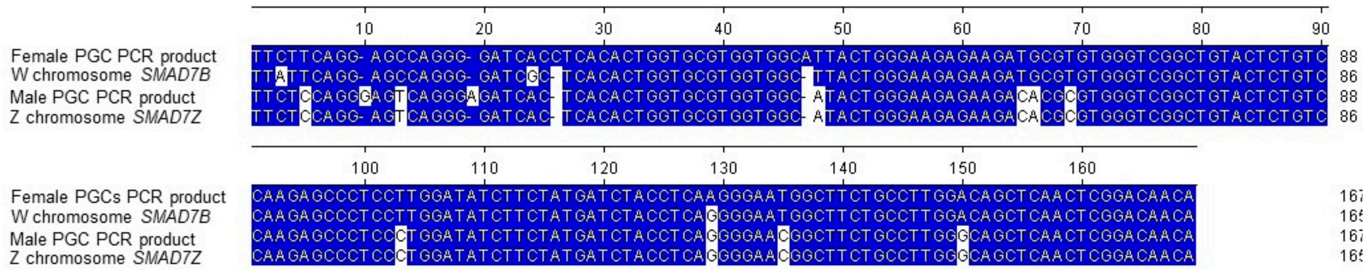

Supplementary Figure S7: The sequence of the amplified *SMAD7* gene obtained by RT-PCR. **A.** RT-PCR shows the amplified PCR products in both male and female PGCs. **B.** Sequencing results of amplified female and male PGC product aligned with the *SMAD7B* gene located on the W chromosome and the *SMAD7Z* gene located on the Z chromosome.

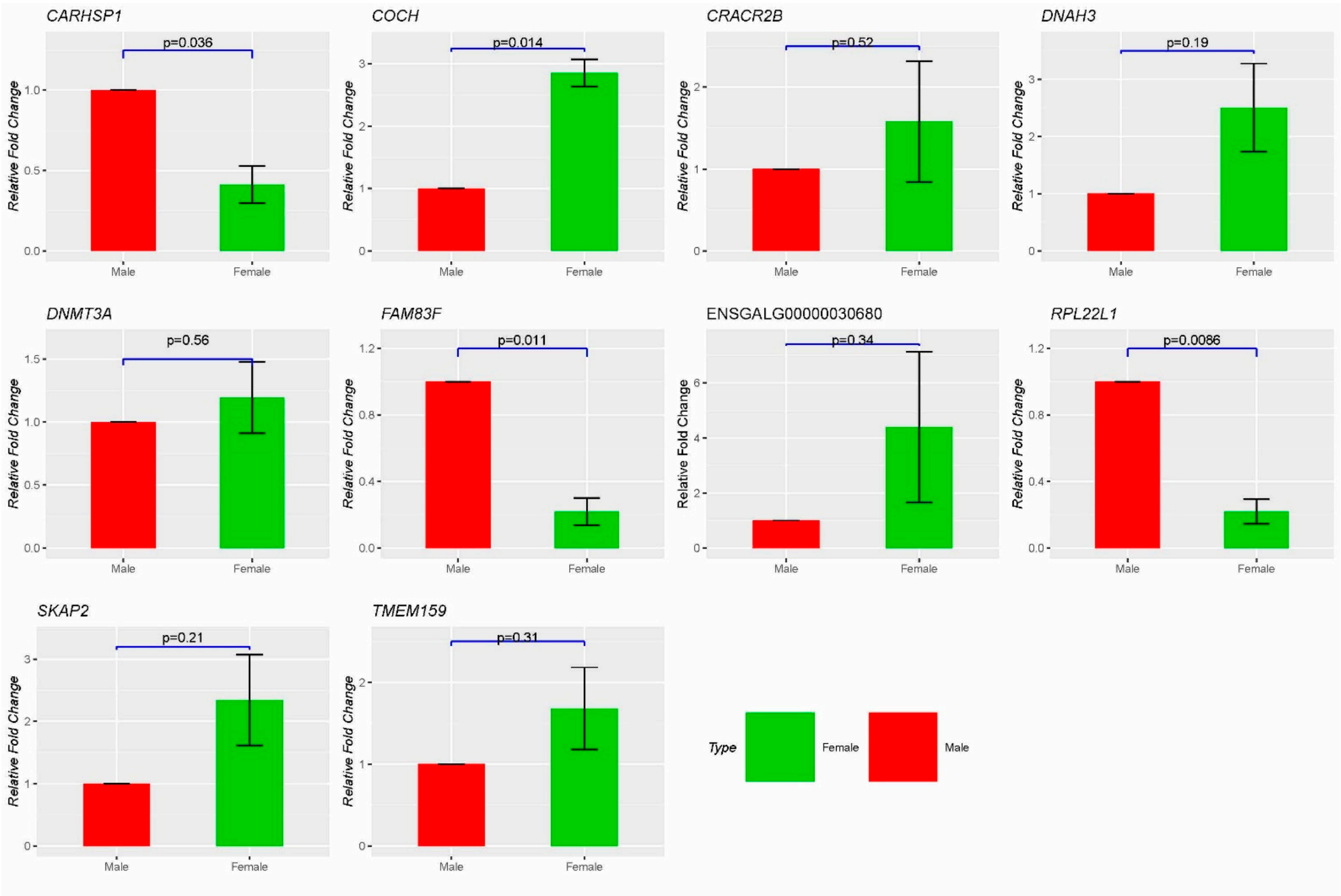

Supplementary Figure S8: RT-qPCR validation of differentially expressed genes in cultured male and female PGCs from a chicken layer breed. The expression of the *COCH* gene is expressed significantly higher in female PGCs with a p-value of 0.014. The genes *FAM83F*, *RPL22L1* and *CARHSP1* are expressed significantly higher in male PGCs (p-value: 0.011, 0.0086 and 0.036 respectively). The genes *SKAP2*, *TMEM159* and *ENSGALG00000030680* were more highly expressed in female PGCs. The genes *CRACR2B* and *DNAH3* are expressed higher in male and female PGCs respectively. The gene *DNMT3A* is expressed equally in both sexes. The expression of a gene in male PGCs was used as a normal level of expression. The p-values are annotated by a blue line and with the corresponding p-value. Three biological replicates were assayed and a p-value lower than 0.05 were considered as significant.

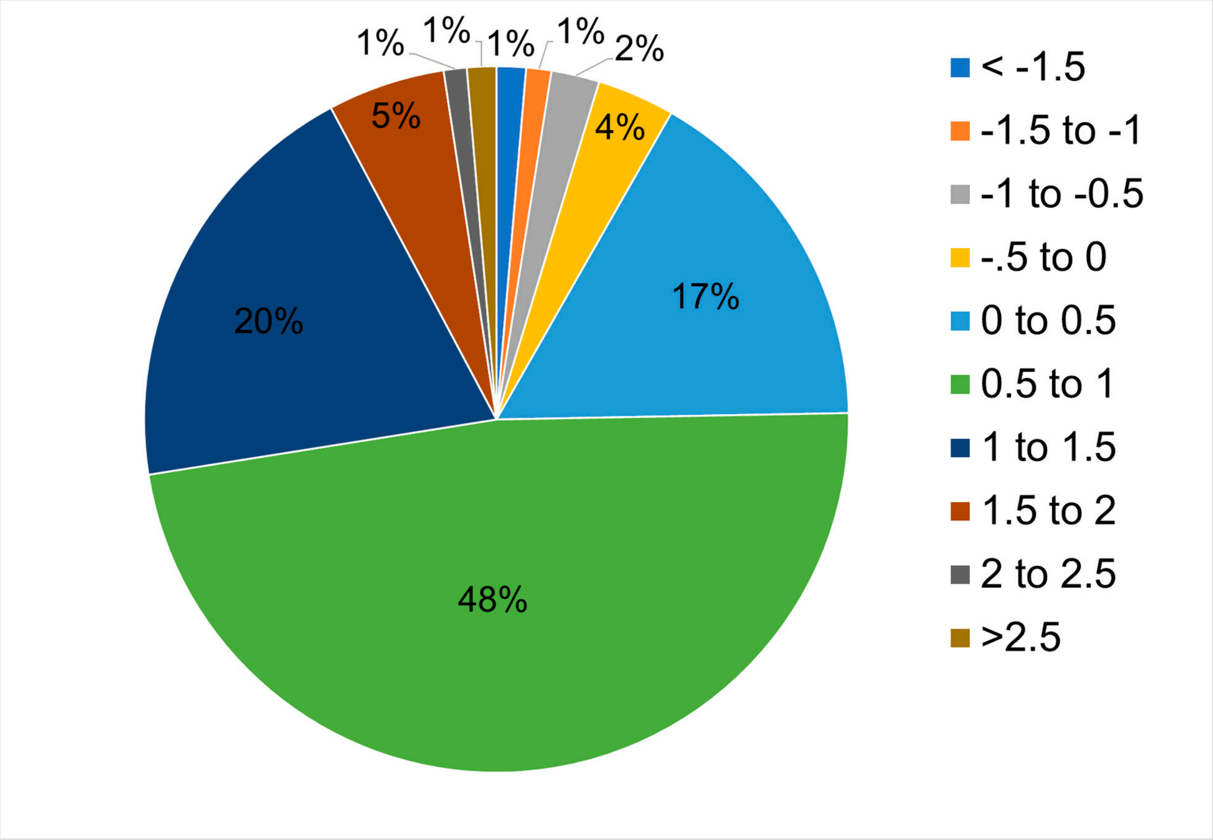

Supplementary Figure S9: Distribution of Log2Foldchange values for Z-linked genes. The pie chart shows the distribution of Log2Foldchange values for Z chromosome-linked genes (m/f). 48% of genes showed log2fold values ranging between 0.5 to 1, 20% of genes have a log2fold value of 1-1.5, 17% of genes have 0-0.5, and only 1% of genes have a log2fold value higher than 2.5. Log2fold values were calculated by the average expression of genes in male cells compared to female cells.

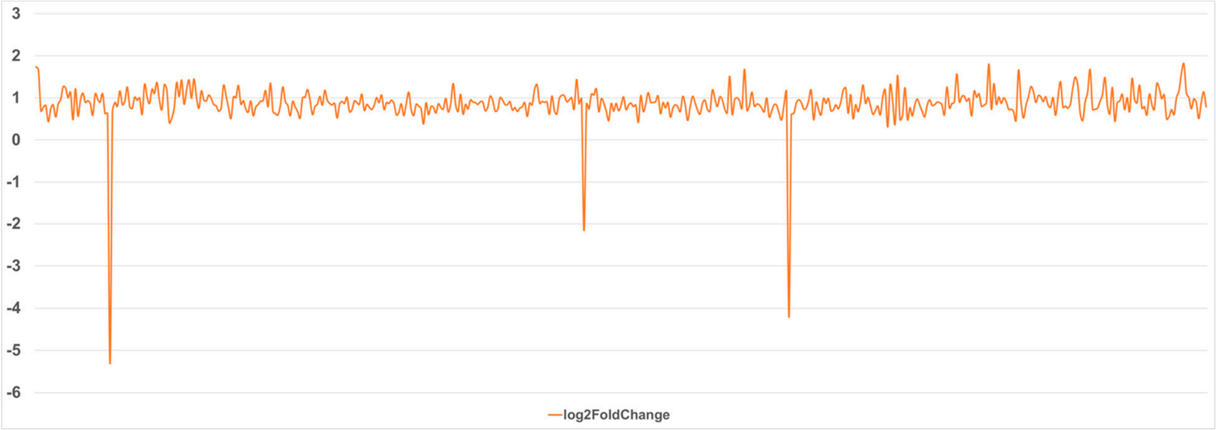

Supplementary Figure S10: Line graph showing log2 fold change values for the Z chromosome-linked genes. The log2fold change values for the genes located on the Z chromosome range between -5 to 1.5.

A

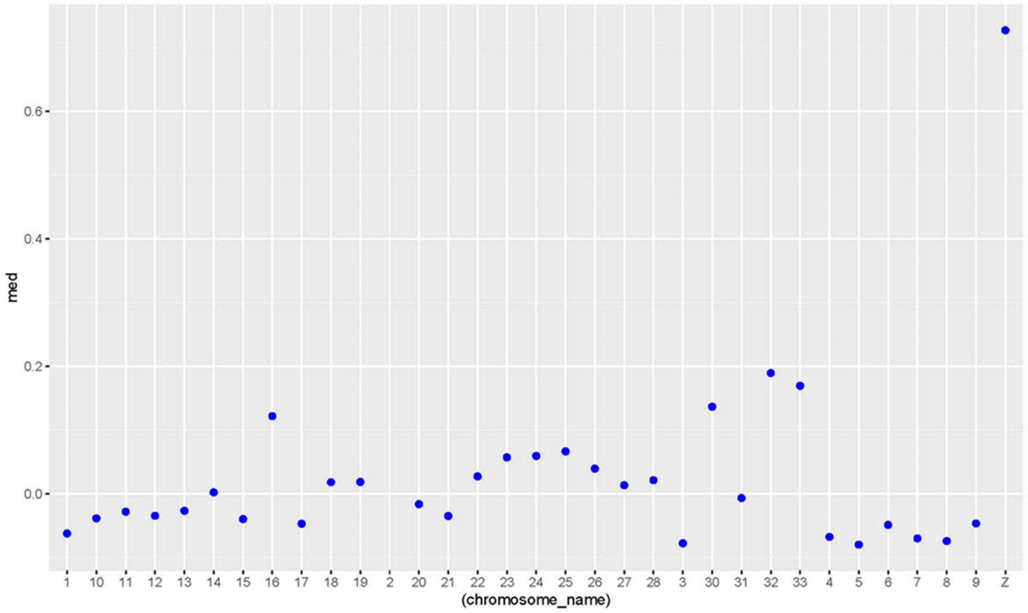

B

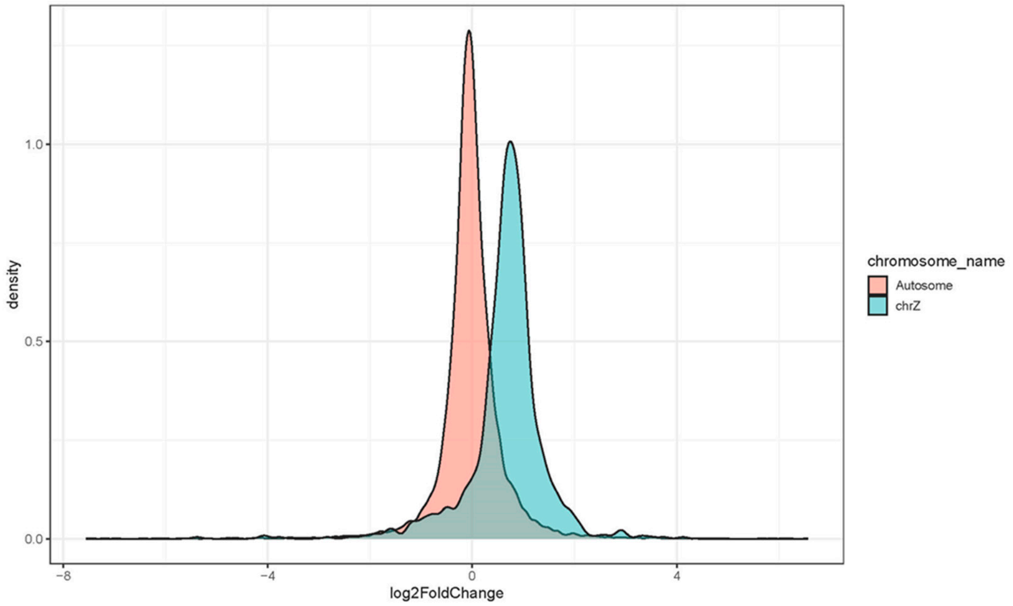

Supplementary Figure S11: A. Chromosome-wise fold change expression of genes from the cultured and *in ovo* PGCs. The median value for the Z chromosome is the highest compared to the other chromosomes. **B.** Comparison of fold change distribution of autosomes and Z chromosome-linked genes from both cultured and *in ovo* PGCs. It shows that Z chromosome-linked genes have higher log2 fold change values than autosomes.

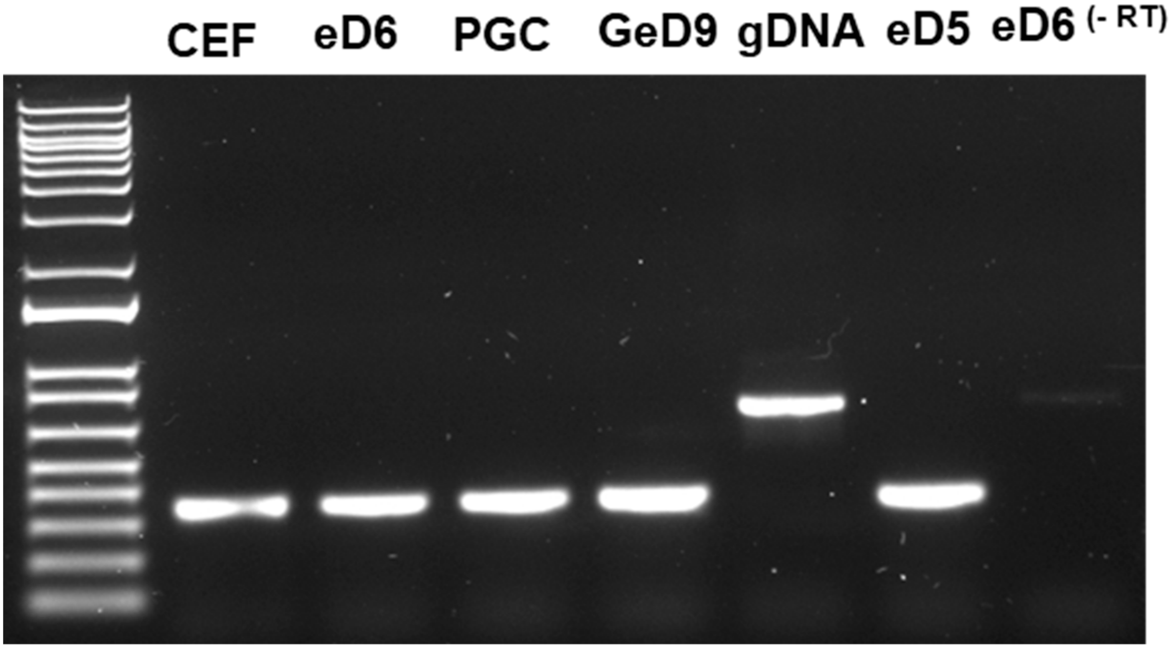

Supplementary Figure S12: RT-PCR analysis for housekeeping gene *GAPDH* on the cDNA isolated from different cell types and tissues. The expected band sizes for *GAPDH* in cDNA and genomic DNA are 350bp and 650bp respectively.
